# Phytochemicals in Cancer Treatment and Cancer Prevention—Review on Epidemiological Data and Clinical Trials

**DOI:** 10.3390/nu15081896

**Published:** 2023-04-14

**Authors:** Anna Rudzińska, Pola Juchaniuk, Jakub Oberda, Jolanta Wiśniewska, Witold Wojdan, Katarzyna Szklener, Sławomir Mańdziuk

**Affiliations:** Department of Clinical Oncology and Chemotherapy, Medical University of Lublin, 20-954 Lublin, Poland; anna.rudzinska05@gmail.com (A.R.);

**Keywords:** beta-carotenoid, cancer, cancer prevention, dietary chemoprevention, flavonoids, phenolic acid, phytosterol, phytochemicals, stilbenes

## Abstract

Phytochemicals are a non-nutritive substances that are present in plants and contribute significantly to their flavor and color. These biologically active compounds are classified into five major groups, namely phenolics, carotenoids, organosulfur compounds, nitrogen-containing compounds, and alkaloids, and are known for their potential health benefits in the prevention of various diseases, including cancer. The purpose of this review article is to explore the potential therapeutic benefits of the dietary phytochemicals, such as flavonoids, phenolic acids, phytosterols, carotenoids, and stilbenes, in cancer treatment and prevention based on the epidemiological studies and clinical trials. Although the majority of epidemiological studies report a significant advantage of the heightened phytochemical consumption and increased serum levels of these compounds, linking increased exposure with a lower cancer risk across most cancer types, these effects could not be replicated in the most available clinical trials. In fact, many of these trials were withdrawn early due to a lack of evidence and/or risk of harm. Despite the strong anticancer effect of phytochemicals, as well as their proven efficacy in multiple epidemiological studies, there is still a great need for human studies and clinical trials, with great caution regarding the safety measures. This review article provides an overview of the epidemiological and clinical evidence supporting the potential chemopreventive and anticancer properties of phytochemicals, with a focus on the need for further research in this area.

## 1. Introduction

Phytochemicals are non-nutritive substances found in plants that contribute significantly to the flavor and color of the plants, as well as the beverages derived from them. They are also being studied for their potential health benefits [1,2]. Some of the potential mechanisms underlying phytochemical health benefits include their role as substrates for biochemical reactions, cofactors of enzymatic reactions, inhibitors of enzymatic reactions, absorbents/sequestrants that bind to and eliminate undesirable constituents in the intestine, ligands that agonize or antagonize cell surface or intracellular receptors, scavengers of reactive or toxic chemicals, compounds that enhance the absorption and or stability of essential nutrients, selective growth factors for beneficial gastrointestinal bacteria, fermentation substrates for beneficial oral, gastric, or intestinal bacteria, and selective inhibitors of deleterious intestinal bacteria [3,4,5,6,7,8,9]. There is a large body of evidence supporting phytochemicals’ effectiveness in the prevention of various diseases, including cardiovascular disease, diabetes, osteoporosis, cataracts, menopausal conditions, gastrointestinal disorders, atopic eczema, hyperactivity, gynecological, neurological, and immunological disorders and even cancer [10,11,12]. According to the data, more than 19.3 million new cancer cases were recently diagnosed and reported, resulting in approximately 10 million deaths by 2020 [13]. Cancer incidences are constantly increasing around the world, resulting in millions of deaths each year, indicating that new approaches to cancer control are urgently needed. An alternative approach to this problem could be to focus on carcinogenesis control rather than attempting to cure the end-stage disease of cancer [14]. Chemoprevention—the use of natural, synthetic, or biologic chemical agents to reverse, suppress, or prevent carcinogenic progression to an invasive cancer—is a possible way to achieve this assumption [15]. It is estimated that an appropriate lifestyle modification could prevent more than two-thirds of human cancers, and diet is responsible for 10–70% (on average, 35%) of the human cancer mortality. Population and laboratory studies are accumulating evidence that phytochemicals have significant anticarcinogenic and antimutagenic properties [16]. Numerous studies on cell lines and animal models have found phytochemicals to be effective in both the treatment and the prevention of cancer, and the results appear to be very promising. It has been demonstrated that phytochemicals extracted from medicinal plants can reduce cell proliferation, cause apoptosis, delay metastasis, and inhibit angiogenesis. Garlic has inhibitory effects on the growth of the Hep-2 human larynx carcinoma cell line, according to the research by Hadjzadeh et al. [17]. Studies on the effect of the *Hibiscus sabdariffa* leaf extract on human cell and xenograft models revealed that it induces autophagic cell death in human melanoma, inhibits the growth of LNCaP in xenograft tumor studies and human cell models, and induces apoptosis in human leukemia and gastric carcinoma cells [18,19,20,21]. Purushothaman et al. discovered in their study using a rat model that Shemamruthaa has a significant anti-cancer effect due to its role in reducing LPO, preventing membrane damage, and restoring membrane integrity [22]. It was demonstrated by Mortazavian et al. that the ethyl acetate and *n*-butanol fractions of *Viola tricolor* have significant antitumor effects against the neuroblastoma N2a cells in their study on a human cell line [23]. The ethyl acetate fraction of *Viola tricolor* has potential cytotoxic properties by reducing tumor cell proliferation, inducing apoptosis, and inhibiting angiogenesis on CAM, according to the research by Sadeghnia et al. [24] Berrington et al. discovered that rosemary species have possible chemopreventive properties because of their high antioxidant content in their research on the anticancer activity of specific herbs and spices [25]. *Vitex rotundifolia* fractions caused apoptosis in a human breast cancer cell line in Chaudhry et al.’s research [26]. HT-29 cell development was inhibited by *Vitex rotundifolia*, according to the research by Jo KJ et al. on the plant’s cytotoxic effects on human colon cancer cell lines [27]. Despite this, there are only a few clinical trials and human studies available [28,29,30,31]. Biologically active phytochemicals are classified into five major groups: phenolics, carotenoids, organosulfur compounds, nitrogen-containing compounds, and alkaloids, all of which have the ability to control carcinogenesis at various stages [32]. In this article, we seek to provide a thorough overview of the literature on the effect of the particular compounds in the field of oncology, as well as a summary of the role of phytochemicals in cancer prevention.

## 2. Methodology 

In light of possible phytochemical-based anticancer treatment, the revelations describing the usage of phytochemicals in the cancer treatment and chemoprevention hold significant value. 

The aim of this review is to evaluate the existing literature on the potential use of phytochemicals as an anticancer agent—both in cancer treatment and prevention. The majority of the existing literature focuses on the complex and multifactorial physiological mechanisms through which phytochemical compounds influence cancer cells in vitro, including oxidative stress, mitochondrial dysfunction, inflammation, and DNA damage [3,4,5,6,7,8,9].

This review is to explore the possible therapeutic benefits of the potential application of phytochemicals in the diet and/or supplementation based on the epidemiological studies and clinical trials. The methodology involved a comprehensive search of the relevant databases (PubMed, Google Scholar), using appropriate keywords related to the theme (phytochemicals/flavonoids/carotenoids/phytosterols/stilbenes/epidemiology/cancer/clinical trials). All identified publications on the influence of phytochemicals on cancer treatment and cancer prevention in humans were included. Data from selected articles have been presented as a classical review. The number of retrieved studies in the compound-specific subsections has been provided in Table 1.

**Figure 1:**
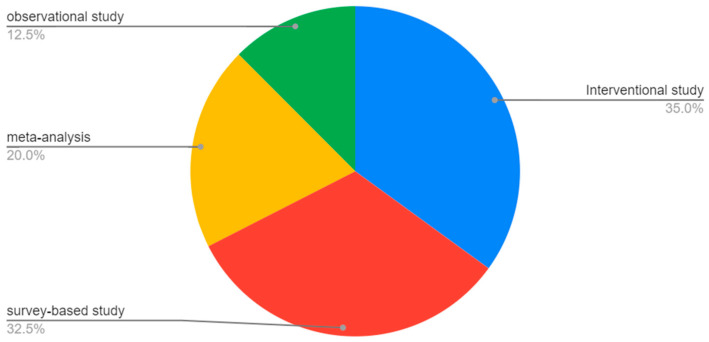
Literature distribution based on the study type.

The main limitation of this review is the relatively small amount of available literature, including interventional studies, survey-based epidemiological studies, meta-analyses, and observational studies (Figure 1).

In addition, the difficulties in the data collection and interpretation caused by the varied research methodology results in the lack of a solid theoretical basis for the assessment of the collected material, which increases the risk of bias. 

Human studies on phytochemical benefits remain the great minority in comparison to the available cell line and animal models in the field of plant-derived substance research. The vast majority of phytochemical human studies included great-sized cohorts and multiple years of study duration, which can be seen in a comparison between case study groups and the control groups (Table 2).

**Figure 2:**
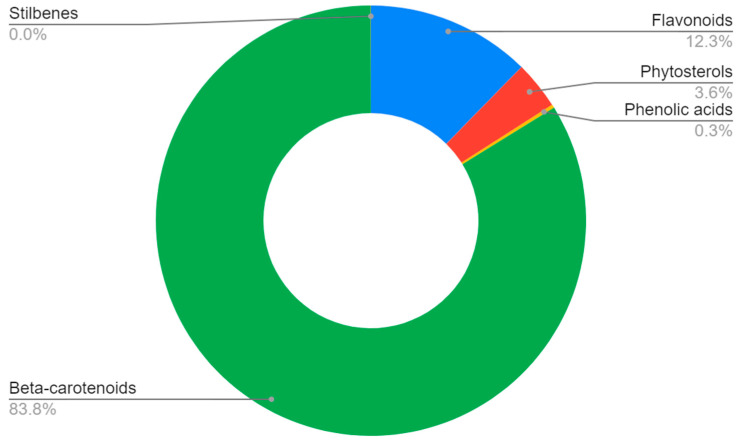
Percentage share of the described studies based on the cohort size.

**Figure 3:**
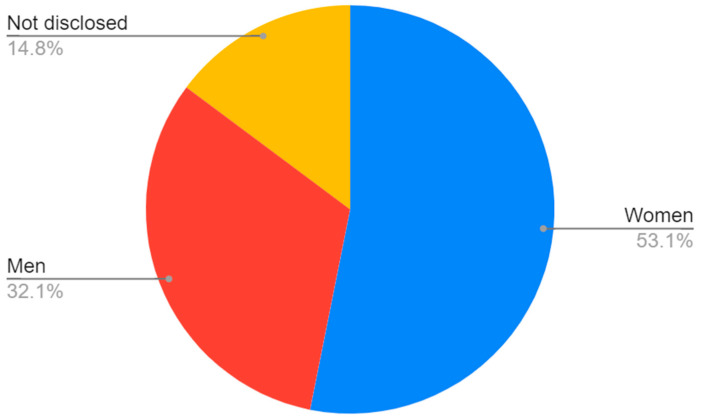
Gender status of the participants of the studies.

**Figure 4:**
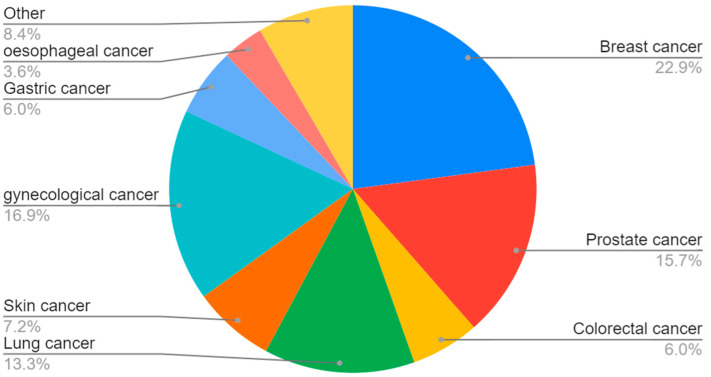
Distribution of the cancer-specific research based on the number of the studies.

The greatest number of patients participated in beta-carotenoid studies, followed by flavonoids (Figure 2). The majority of the participants were identified as women, although this is strongly linked to the great number of studies on breast cancer (Figure 3 and Figure 4). 

## 3. Results 

### 3.1. Flavonoids

The flavonoids in plants are derived from the aromatic amino acids phenylalanine and tyrosine, as well as acetate units, and participate in the light-dependent phase of photosynthesis, catalyzing electron transport [33]. They constitute the majority of polyphenolic secondary plant metabolites found in human diets [34]. Fruits (particularly citrus and berries), nuts, and vegetables (such as tomatoes, cabbage, onions, and beans), as well as beverages, such as tea, tisanes (herbal teas), and red wine, are excellent sources of dietary flavonoids. 

Flavonoids are not very bioavailable, with only about 10% of the amount consumed reaching peak concentrations in the circulation in the nanomolar or low micromolar range after a few hours [35,36,37]. It is estimated that the daily consumption for humans ranges from 20 to 70 mg/d and may even be as high as 500 mg/d [34]. 

Flavonoids have received a great deal of attention due to their numerous pharmacological properties, including anti-cancer activity. The most important antitumor properties of flavonoids are based on their abilities as anti-inflammatory and antioxidant compounds [38,39]. The flavonoid structure’s numerous hydroxy groups, combined with a highly conjugated electron system, allow them to act as a free radical scavengers via hydrogen atom or electron-donating activities. They can also inhibit the formation of ROS and act as pro-oxidative compounds [40]. 

**Figure 5:**
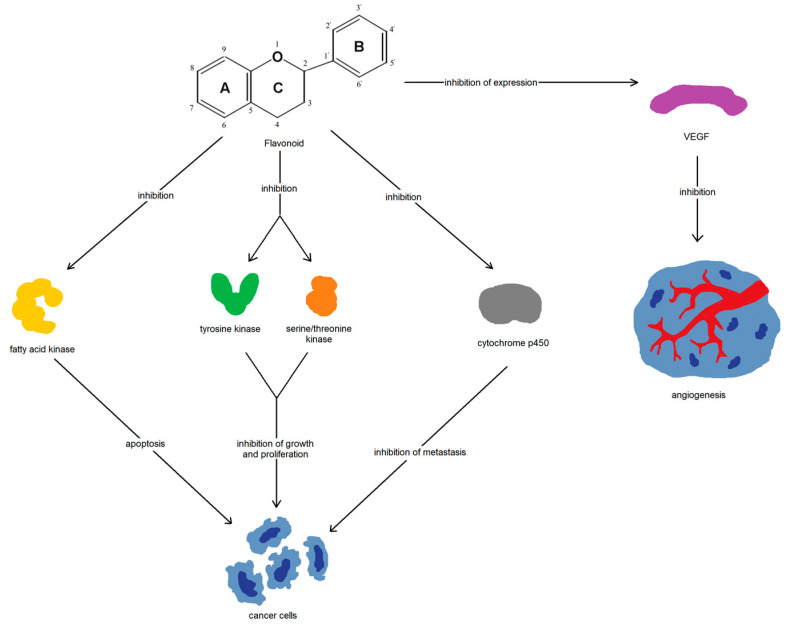
Anticancer mechanism of action of the flavonoid.

Flavonoids have an anti-inflammatory effect by inhibiting the nuclear translocation, expression, or phosphorylation of the transcription factors involved in the inflammatory process (Figure 5). There is evidence that they can modulate the nuclear factor B (NF-B), mitogen-activated protein kinase (MAPK), focal adhesion kinase (FAK), PI3K-Akt, inflammasome, and signal transducer and activator of transcription 3 (STAT3) pathways [41,42]. Flavonoids have also been linked to the induction of apoptosis due to their ability to inhibit fatty acid synthase activity [43]. Furthermore, they demonstrate the inhibition of tyrosine and serine/threonine kinases, impacting cancer cell growth and their proliferation [44]. Other significant anti-cancer mechanisms of flavonoids include modulation of the cytochrome P450 and phase-II enzymes, inhibition of angiogenesis through the inhibition of vascular endothelial growth factor (VEGF) expression, and inhibition of metastasis via a reduction in MMP protein expression and the induction of autophagy [45,46,47,48,49].

Many epidemiological studies have shown an inverse association between the risk of cancer development and the consumption of flavonoid-rich diets, especially for breast, gastric, lung, and esophageal tumors, among others [50,51]. 

#### 3.1.1. Flavonoids and Breast Cancer

One of the early case-control studies on the association between phyto-estrogen intake and the risk of breast cancer by Ingram D. et al. showed a substantial reduction in breast cancer risk among women with a high intake of the phyto-estrogens—particularly the isoflavonic phyto-estrogen equol and the lignan enterolactone. Phyto-estrogen intake was measured based on urinary excretion, and 144 samples were included for the analysis. The findings were claimed to have potential for the prevention of breast cancer [52]. Another study, investigating 250 urine samples collected from Chinese women in Shanghai, revealed a reduction of the risk of breast cancer with increasing excretion of total isoflavonoids and total lignans. The adjusted odds ratio was 0.28 (95% confidence interval, 0.15–0.50) for women who had a high excretion rate of both total lignans and isoflavonoids compared with those with low excretion of both groups of phytoestrogens. The results further confirm that the high intake of certain phytoestrogens may reduce the risk of breast cancer [53]. The association between flavonoid intake and the reduced breast cancer risk was also suggested in a case-control study conducted among women who resided in Nassau and Suffolk counties in Long Island, New York. Cases (*n* = 1434) and controls (*n* = 1440) were interviewed about the known and suspected risk factors and asked to complete a food frequency questionnaire regarding their average intake in the prior 12 months. The decrease was most pronounced among postmenopausal women for flavonols, flavones, flavan-3-ols, and lignans [54]. J Peterson et al. found a strong, statistically significant inverse association of flavone intake with breast cancer in a large case-control study of 820 women with breast cancer and 1548 control women, conducted in Greece. The inverse association of flavones implies a 13% reduction in the breast cancer risk per 1 s.d. (0.5 mg day^−1^) of an increase in the intake of the respective compounds. Inverse associations with breast cancer risk were also found for flavonols, flavan-3-ols, and anthocyanidins [55]. Ying Wang et al. examined the associations between seven subclasses of dietary flavonoids and the invasive postmenopausal breast cancer risk overall and by ER status in a U.S. prospective cohort of 56,630 postmenopausal women, among whom 2116 invasive breast cancers were verified during a follow-up. There was a modest inverse association between flavone intake and the overall breast cancer risk and between flavan-3-ol intake and risk of ER− breast cancer but not for ER+ breast cancer risk. Those results also seem to be consistent with the above-mentioned studies indicating a beneficial role of plant-based diets in breast cancer risk [56]. 

Several meta-analyses show an association between soy isoflavone intake and the reduction of breast cancer risk [57,58,59]. Hui et al.’s study, involving 9513 cases and 181,906 controls, six of which were prospective cohort studies and six were case-control studies, implies that the intake of flavonols and flavones, but not other flavonoid subclasses or total flavonoids, is associated with a decreased risk of a breast cancer, especially among post-menopausal women [60]. Moreover, a meta-analysis including five cohort studies (11,206 patients) revealed that soy food intake might be associated with reduced mortality and recurrence, especially for ER-negative, ER+/PR+, and postmenopausal patients [61]. 

#### 3.1.2. Flavonoids and Prostate Cancer 

The case control study by Lee MM et al. in China based on 133 cases and 265 age- and residential community-matched controls evaluated the effect of the soy isoflavone intake on the risk of prostate cancer with the result suggesting a reduction of this risk associated with the consumption of soy products [62]. A population-based prospective study on 43,509 Japanese men showed similar findings [63]. Milan S. Geybels et al. investigated the associations among flavonoid intake, black tea consumption, and prostate cancer risk in the Netherlands Cohort study, which included 58,279 men. From 1986 to 2003, 3362 prostate cancers were identified, including 1164 advanced (stage III/IV) cancers. Results implied an association between dietary flavonoid intake and a decreased risk of advanced-stage prostate cancer [64]. A case control study nested in the European Prospective Investigation into Cancer and Nutrition indicates that higher plasma concentrations of the isoflavone genistein, but not other isoflavones, were associated with a lower risk of prostate cancer [65]. The University of Hohenheim a conducting the randomized controlled double-blind crossover trial (NCT01538316), which aimed to compare the effects of the flavonoid quercetin and isoflavone genistein with those of a placebo on the rate of increase in prostate-specific antigen (PSA). At the beginning of the study in March 2012, 60 participants were recruited. Over a period of 18 months, the primary outcome was determined by the increase in PSA every three months. The study’s completion date was projected to be April 2014, but the recruitment process unfortunately has not been updated since 15 May 2012. Meta-analyses of the two studies, including men with an identified risk of PCa, found a significant reduction in the PCa diagnosis after the administration of soy/soy isoflavones, and therefore, there may be support for the epidemiological findings of a potential role for soy/soy isoflavones in PCa risk reduction [66]. 

#### 3.1.3. Flavonoids and Gastrointestinal Cancer 

The European Prospective Investigation into Cancer and Nutrition (EPIC) study looked at the link between dietary flavonoids, lignans, and gastric cancer incidence (GC). The study by Zamora-Ros et al. included 477,312 subjects (29.8% men) aged 35–70 years from ten European countries, with 683 incident GC cases confirmed over an 11-year period. Total flavonoid intake was found to have a significant inverse relationship with GC risk in women but not in men [67]. Similar results were obtained by Woo, H.D. et al. in their case-control study on the association between GC and flavonoid intake in the Korean population. The study conducted with 334 cases and 334 matched controls aged 35–75 years suggested a significant inverse association between the gastric cancer risk and dietary flavonoids and their subclasses, with the exception of anthocyanidins and isoflavones. Also in this case, the above-mentioned effects were observed in women, but not in men. Moreover, no significantly different effects were observed in the subgroup analysis of H. pylori and smoking status [68]. Petrick J. et al. investigated the relationship between flavonoid intake and the incidence and the survival of esophageal and gastric cancer in a multicenter, population-based study in the United States. Case participants with the esophageal adenocarcinoma (*n* = 2740), gastric cardia adenocarcinoma (*n* = 248), esophageal squamous cell carcinoma (*n* = 191), and other gastric adenocarcinomas (*n* = 341) were followed until 2000 for vital status, and 662 frequency-matched controls were included in the study. The total flavonoid intake had little or no consistent association, but the anthocyanin intake was associated with a 57% reduction in the risk of incident esophageal adenocarcinoma and a lower risk of mortality from gastric cardia adenocarcinoma [69]. Zamora-Ros et al. looked at the link between dietary flavonoid and lignan intake and the risk of colorectal cancer in a Spanish population. The study included 424 colorectal cancer cases and 401 hospital-based controls. The findings indicated an inverse relationship among total flavonoids, lignans, some individual flavonoid subgroups, and the risk of colorectal cancer [70]. 

#### 3.1.4. Flavonoids and Lung Cancer 

Le Marchand et al. conducted a population-based, case-control study in Hawaii to investigate the possible relationship between flavonoid intake and a lung cancer risk. The study included 582 patients with incidental lung cancer and 582 healthy controls. An in-person interview was conducted to assess the smoking history and typical intake of 242 food items. The findings indicated statistically significant inverse associations between lung cancer risk and the main food sources of the flavonoids quercetin (onions and apples) and the naringin (white grapefruit). Furthermore, the effect of onions on squamous cell carcinoma was modified by the CYP1A1 genotype, implying that the CYP1A1 may play a role in this association [71]. Cui Y. et al. conducted another population-based case-control study involving 558 lung cancer cases and 837 controls to investigate the associations between commonly consumed flavonoid compounds and a lung cancer. Flavonoid dietary intake was calculated by combining intake frequency (as determined by a food frequency questionnaire), the portion size, and the food composition data. Lung cancer was found to be inversely related to epicatechin, catechin, quercetin, and kaempferol consumption in smokers but not in nonsmokers. There was little association with total flavonoids, including the thearubigins, hesperetin, naringenin, and myricetin, regardless of the smoking status [72]. The analysis of data from a case-control study of lung cancer in women in Spain (103 cases and 206 hospital controls) to assess the relationship with the intake of the specific carotenoids and flavonoids (luteolin, kaempferol, myricetin, and luteolin) revealed no association for luteolin and quercetin and only a nonsignificant association for the highest vs. lowest tertile intake of kaempferol. There was also no correlation with total flavonoid intake [73]. The prospective population-based Kuopio Ischaemic Heart Disease Risk Factor (KIHD) Study, which included 2590 Finnish men, discovered that the two of the five flavonoid subclasses examined, flavonols and flavan-3-ols, were associated with a lower risk of lung cancer [74]. In a large prospective study of 34,708 postmenopausal women, the Iowa Women’s Health Study looked at the relationship between total flavonoids and seven flavonoid subclasses and the risk of lung, colorectal, breast, pancreatic, and upper aerodigestive cancer. Flavonoid intake was estimated from three databases developed by the USDA Nutrient Data Laboratory (NDL). The findings revealed that the consumption of flavanones and proanthocyanidins was inversely related to lung cancer incidence in the current and former smokers, but not in the never smokers [51]. A case-control study in Uruguay establishing the relationship between dietary antioxidants and lung cancer risk found that quercetin and total flavonoid intake reduced the risk significantly, but kaempferol had no effect. [75]. The combined findings of the meta-analysis, which included eight prospective studies and four case-control studies involving 5073 lung cancer cases and 237,981 non-cases, demonstrated a statistically significant correlation between the highest flavonoid intake and a reduced risk of developing lung cancer. Furthermore, a 20 mg/day increase in flavonoid intake was related to a 10% lower risk of developing lung cancer [76]. 

### 3.2. Phytosterols 

Phytosterols are the natural products that exist in plants in both esterified and free alcohol forms, and the highest concentration of them can be found in vegetable oils, cereal grains, cereal-based products, nuts, legumes, vegetables, and fruits, with the largest amount in vegetable oils [77,78,79,80,81]. 

To the most common phytosterols and phytostanols belong sitosterol, sitostanol, campesterol, campestanol, and brassicasterol. Phytosterols have been proven to reduce blood cholesterol levels by reducing the intestinal absorption of cholesterol. They are also suggested to offer protection against a variety of chronic ailments, including cardiovascular diseases, obesity, diabetes, and cancer [77,79]. 

**Figure 6:**
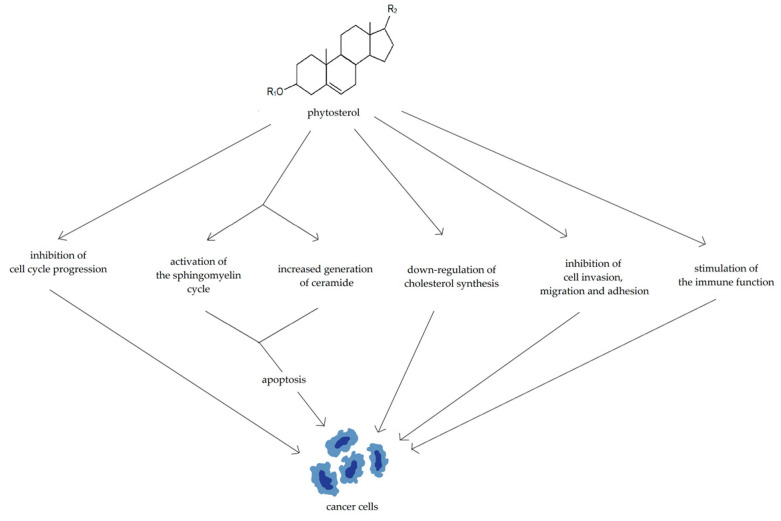
Anticancer mechanism of phytosterols.

Various mechanisms have been proposed to explain the potential anticancer properties of phytosterols (Figure 6): inhibition of cell cycle progression, promotion of cellular apoptosis possibly via activation of the sphingomyelin cycle and increased generation of ceramide, down-regulation of cholesterol synthesis, inhibition of cell invasion, migration, and adhesion, and stimulation of the immune function [78]. 

Vegetarians tend to have lower overall rates of cancer than the general population [82]. The administration of phytosterols in a diet has been proven to lower the risk of multiple cancer risk factors, including obesity, diabetes, hypertension, and serum CRP levels [83,84,85,86]. 

Various in vitro and in vivo studies imply the strong anticancer modality of phytosterols, which has been researched using various cancer cell lines and animal models [87,88,89,90,91]. 

#### 3.2.1. Phytosterols in Breast and Gynecological Cancers 

In a series of studies conducted in Uruguay, the protective effect of plant sterol intake on breast and ovarian carcinogenesis was reported [92]. Two investigations conducted in New York supported an association between reduced ovarian and endometrial cancer risks and a diet high in plant foods, which included phytosterols [93]. Similarly, the naturally occurring dietary intake of phytosterols in a Spanish free-living population has been also estimated to be higher than that in people living in other non-Mediterranean European countries, suggesting that this could be a part of the Mediterranean diet phenomenon. A randomized, clinical trial conducted in 2015 in Spain involving a Mediterranean diet intervention showed a reduction in the breast cancer risk in the intervention group [94]. The hospital-based case-control study conducted in Mexico City between 1994 and 1996 supports a protective role of specific dietary phytochemicals in breast cancer risk by menopausal status, independent of other reproductive factors [95].

A clinical trial, conducted in 2020 by the University of Leeds, aimed to establish the influence of LDL-C-lowering dietary intervention on the ability of non-cancer cells (adipocytes, fibroblasts, and macrophages) to change the chemotherapy response and the metastatic process in breast cancer cells. The main intervention of this study involved supplementation with phytosterols in the form of an enriched yoghurt to the volunteers with high LDL-C levels over the course of 8 weeks. To evaluate the effectiveness of the supplementation, volunteers’ blood, white blood cells (macrophages), and fat tissue cells were examined with the purpose of measuring oxysterol, LDL-C, and phytosterol concentrations and to measure alterations in the behavior of cancer cells mediated by the host cells in the laboratory examination. The status of the study remains as recruiting, and the last update was posted on 20 May 2021 [96]. 

#### 3.2.2. Phytosterols in Prostate Neoplasms

A study by Fraser in 1999 showed that vegetarians have a lower risk of prostate cancer—54% greater in vegetarians (*p* ≈ 0.03) than in the nonvegetarians. A multivariate evaluation revealed an association of chemoprevention with higher dried fruit intake (*p* < 0.05) [83]. 

#### 3.2.3. Phytosterols in Colorectal Cancer 

Despite the chemoprotective model of dietary phytosterols in animal studies [77,78,79], a high dietary intake of the plant sterols was not associated with a lower risk of colon and rectal cancers in the Netherlands Cohort Study on Diet and Cancer [97]. Chinese research by Jing Huang et al. in 2017 indicated that the consumption of total phytosterols, β-sitosterol, campesterol, and campestanol is inversely associated with colorectal cancer risk in a Chinese population [98]. A study conducted by Fraser from 1999 on the Seventh-day Adventists population claimed that the risk of a colon cancer was increased by 88% in nonvegetarians compared with that in vegetarians (*p* < 0.003). Increased legume consumption over three times a week was associated with a much lower relative risk of colon cancer (0.33; 95% CI: 0.13, 0.83), but only in the red meat-eating nonvegetarians [83]. 

#### 3.2.4. Phytosterols in Lung Cancer

In a case-control study conducted in Uruguay in 1993–1996 and including 463 cases of lung cancer and 465 hospitalized controls, the influence of phytosterols on a neoplasm was evaluated. Total phytosterol intake was inversely correlated with lung cancer risk (OR 0.50, 95% CI 0.31–0.79) and the dose–response effect was statistically significant (*p* = 0.002). The relationships between beta-sitosterol, campesterol, and stigmasterol and the lung cancer risk were similar. This protective effect was distinctly evident for adenocarcinoma of the lung [99]. Fraser established a strong inverse association between fruit consumption and the risk of lung cancer in the Seventh-day Adventists population for both main histologic subtypes [100]. 

#### 3.2.5. Phytosterols in Stomach Cancer 

Between 1997 and 1999, a case-control study involving 120 cases of stomach cancer and 360 controls was conducted in Uruguay. Total phytosterols were associated with a strong inverse relationship with stomach cancer. Combined exposure to the high intake of total phytosterols and alpha-carotene was also inversely associated with the gastric cancer risk [101]. 

#### 3.2.6. Phytosterols in Pancreatic Cancer 

Mills et al.’s research implies a significant negative association between the consumption of dried fruits (*p* < 0.05), legumes (*p* = 0.01), and also vegetarian meat analogues (*p* = 0.03) and pancreatic cancer risk in the Seventh-day Adventists population [102]. 

### 3.3. Phenolic Acid 

Phenolic acids belong to polyphenols and are divided into hydroxybenzoic acids and hydroxycinnamic acids. The main hydroxybenzoic acids are gallic, ellagic, 4-hydroxybenzoic, and vanillic acid. The most common hydroxycinnamic acids are caffeic, ferulic, p-coumaric, and sinapic acid. Both acid subtypes are widely distributed among foods, such as coffee, berries, nuts, tea, wine, olive, pome, and drupe fruits and vegetables. Highest concentrations of hydroxybenzoic acids were found in chestnuts and highest concentrations of hydroxycinnamic acids were found in coffee [103,104]. 

Moreover, phenolics and polyphenolics comprise a vast array of the biologically active compounds ubiquitous in plants, many of which have been used in Chinese traditional medicine for thousands of years [105]. 

**Figure 7:**
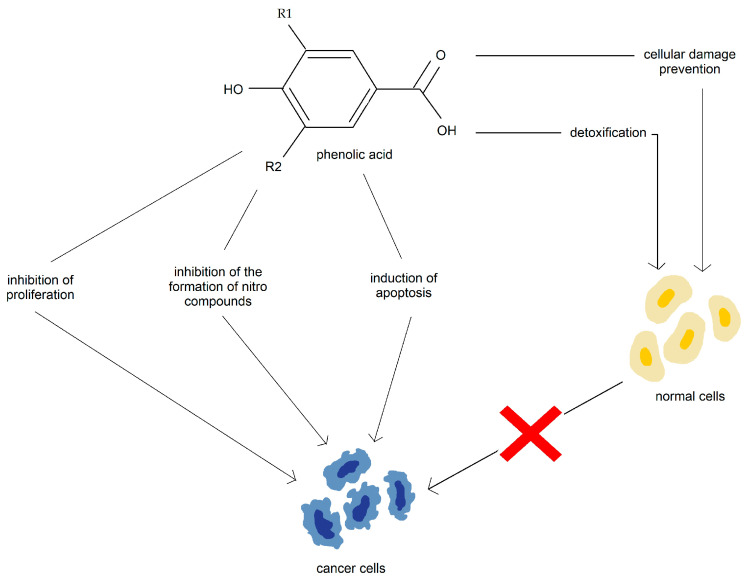
Anticancer mechanism of action of phenolic acid.

The potential anticancer properties of phenolic acids include detoxification, cellular damage prevention, inhibition of cell proliferation, inhibition of the formation of the nitro compounds, and induction of apoptosis (Figure 7) [106,107,108,109,110].

Furthermore, studies suggest that the pro-oxidant action of caffeic acid and ferulic acid, instead of the antioxidant action, might be crucial for both the anticancer effects and the apoptosis-inducing properties of these compounds [106,107,108,109,110].

Studies show that the combination of phenylpropanoids with 5-fluorouracil and ferulic acid with cisplatin or carboplatin can improve cancer treatment [106,107,108,109,110]. Synthetic caffeic acid analogs are also reported to possess anti-inflammatory, anti-cancer, and anti-HIV activities [111]. 

#### 3.3.1. Phenolic Acid in Prostate Cancer 

Results from a population-based case-control study from January 2015 to December 2016 in a single institution of the municipality of Catania, southern Italy, in which 340 patients took part, suggested that high intake of caffeic acid and ferulic acid may be associated with a reduced risk of prostate cancer (PCa) and may have beneficial effects in reducing the PCa incidence [112]. 

#### 3.3.2. Phenolic Acid in Breast Cancer 

In a multipurpose, prospective cohort study of 10,812 middle-aged women who were university graduates conducted by the Seguimiento Universidad de Navarra, findings suggested that the phenolic acid content of the diet, particularly dietary hydroxycinnamic and the chlorogenic acids present in coffee, fruits, and vegetables, was associated with a lower breast cancer risk among the postmenopausal women in this Mediterranean cohort [113]. 

#### 3.3.3. Phenolic Acid in Lung Cancer 

An open-label, randomized controlled trial conducted by Guangzhou University of Chinese Medicine evaluated the efficacy and safety of traditional Chinese medicine (TCM) in advanced non-small cell lung cancer (NSCLC) patients who underwent chemotherapy. One of the types of medicine administered was ZhenqiFuzheng (ZQFZ) capsules consisting of sinapinic acid, ferulic acid, asiatic acid, pratensein, and glycitein. The results from this study showed that TCM treatment could improve the quality of life of NSCLC patients and alleviate their symptoms with good safety [114]. Another Chinese study conducted in 2020 by Yanqing Zhou and Chenxi Wu indicated that ZQFZ granules could treat NSCLC through multitargets and multipathways [115]. 

#### 3.3.4. Phenolic Acid in Skin Cancer

The study conducted by Duke University Medical Center showed that the use of a stable topical formulation of 15% L-ascorbic acid, 1% alpha-tocopherol, and 0.5% ferulic acid was particularly effective for reducing the thymine dimer mutations known to be associated with a skin cancer. However, the relatively small number of patients evaluated limited this study [116].

#### 3.3.5. Phenolic Acid in Esophageal Cancer 

The First Affiliated Hospital of Henan University of Science and Technology registered two clinical trials aiming to investigate the efficiency and safety of caffeic acid for Chinese advanced esophageal squamous cell cancer (ESCC). Unfortunately both studies (2017, 2020) were last updated in 2020, with their status remaining as unknown. The first study involved 240 patients with an advanced ESCC diagnosis split into control and experimental groups, which was to receive 300 mg of the caffeic acid orally until the occurrence of the progression of disease, death, or unacceptable adverse effects. Results were to be assessed after a 1-year follow-up period. The 2020 study aimed to investigate the efficacy of the oral administration of 100–200 mg of caffeic acid, depending on body mass, to 80 advanced ESCC patients who failed chemotherapy or chemoradiotherapy. Treatment was to be administered in a two-week period followed by a one-week black interval. Similarly, the follow-up period was supposed to last 1 year. No results of both studies are available [117,118].

### 3.4. Stilbenes 

Natural stilbenes are a group of the polyphenolic phytochemicals that contain a 1,2-diphenylethylene nucleus and are known for their potential in preventing and treating various diseases, including cancer [119]. Natural polyphenols are plentiful in fruits, vegetables, whole grains, and foods and beverages derived from them, such as chocolate, wine, olive oil, or tea, thus becoming the most important among all phytochemicals present in the human diet [120]. Potential anticancer properties have been suggested for various polyphenols, including, for example, green tea polyphenols, grape seed proanthocyanidins, resveratrol, silymarin, curcumin, quercetin, luteolin, and genistein [120,121]. 

These compounds possess antioxidant, anti-inflammatory, and cell death-activation properties that make them highly beneficial for human health [122,123]. However, despite their numerous benefits, the biological activity of these compounds is still not completely understood, and they are rapidly metabolized when administered to animals or humans [119], which may alter their pharmacokinetics and/or activities. 

**Figure 8:**
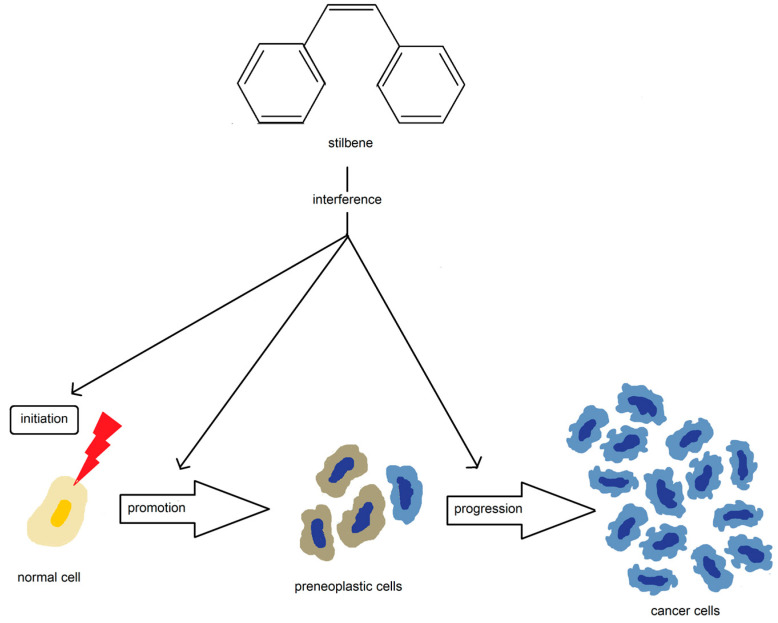
Anticancer mechanism of action of the stilbenes.

Stilbenes have shown the ability to reduce the incidence of tumorigenesis at all stages of carcinogenesis and are known to interfere with the molecular events during the initiation, promotion, and progression of cancer (Figure 8) [124]. 

However, the partial understanding of their mechanisms of action [125] and the low bioavailability [126] due to their chemical structure [127,128,129] remain a challenge. 

Novel drug formulations have been suggested to improve the stability and bioavailability and minimize side effects, in order to make stilbenes more effective candidates for anticancer drugs [130]. 

Among the more than 400 natural stilbenes, resveratrol, pterostilbene, piceatannol, and pinosylvin are the most common [131,132]. Resveratrol, in particular, is the most studied stilbene and has wide-ranging effects that include cardioprotective, anti-oxidative, anti-inflammatory, estrogenic/anti-estrogenic, and anti-tumor properties [133]. It has been found to be useful in managing cognitive disorders and provides considerable protection against cancer induction in various tissues—helping with protection against the initiation of cancer within the oral cavity [134] and the esophagus [135], among other tissues. Its cancer chemopreventive activity aside, resveratrol can also prevent the development and/or cause the regression of established tumors in xenograft models of cancers of the ovaries [136], urinary bladder [137], stomach [138], and head and neck [139,140]. 

Resveratrol treatment effectively suppressed the growth rate of neuroblastoma through the up-regulation of cyclin E and the down-regulation of p21 [141] and also showed antiproliferative and pro-apoptotic properties that make it an excellent antitumor agent [142]. However, most of these effects were observed in vitro or with small animals and were obtained at concentrations that are too high to be compatible with its low availability in vivo [143]. 

Nonetheless, resveratrol is a non-toxic compound and can be used as a food supplement. Its synergistic effect with other food polyphenols and its metabolites’ antiproliferative activities are relevant for diet-dependent cancer prevention [134,144].

#### 3.4.1. Stilbenes and Colorectal Cancer 

The Wnt signaling pathway is often activated in colon cancers, and resveratrol has been shown to modulate this pathway [145]. To further understand the actions of resveratrol on the Wnt signaling pathway, a clinical trial was conducted with colon cancer patients receiving treatment with resveratrol, while laboratory studies examined its effects on colon cancer and the normal colonic mucosa [146]. In a study from 2009, eleven patients were treated with resveratrol in a two-week course in the form of pills (20 mg) or freeze-dried grape extract before undergoing standard surgical resection of the tumor. The dose of resveratrol was gradually increased for each patient (for the first and second patient—a dose of 20 mg/day, the third and fourth—80 mg/day, for the fifth and sixth—160 mg/day plus grape extract at a dose of 125 mg/day mixed with an 8 oz glass of water), and no dose adjustments were made during the trial. Gene expression analysis of the colon cancer tissue showed that some genes (*myc* and *cyclinD1*) increased following exposure to resveratrol or the grape extract, but the mechanism behind these increases is unclear and requires further investigation. The effects of resveratrol at higher concentrations obtained with the pharmacologic doses may be different than those seen in this study [147]. 

#### 3.4.2. Stilbenes and Neuroendocrine Cancer 

Resveratrol can activate a protein called Notch-1, which has been shown to prevent tumor cell growth. A study conducted by the University of Wisconsin, aimed to investigate the effects of resveratrol and Notch-1 on neuroendocrine tumor tissue patients with neuroendocrine tumors, assessed how well they tolerated the product when taken for up to three months. Resveratrol was administered at a dose of 5 gm/day orally, in two divided doses of 2.5 mg each, without stopping for a total of three cycles. The researchers’ primary goal was to demonstrate that resveratrol treatment in patients with low-grade GI neuroendocrine tumors will significantly increase Notch1 activation in post-treatment tumor biopsy specimens when compared to pretreatment levels. The primary endpoint will be the level of expression of full-length Notch1, cleaved Notch1, HES-1, and ASCL-1, as measured by Western blotting using quantitative densitometry [148].

#### 3.4.3. Stilbenes and Breast Cancer 

Loma Linda University has been conducting a study since 2019 [149], which involves 50 participants and aims to investigate the impact of resveratrol on IGF-II levels in healthy African American women. Preclinical studies have demonstrated that resveratrol inhibits IGF-II and promotes apoptosis in the breast cancer cell lines [149]. The researchers intend to assess the baseline IGF-II levels in healthy African American women, who are at a higher risk of mortality and worse outcomes when treated with the standard adjuvant therapies for the breast cancer. The study will monitor the IGF-II levels of participants receiving the resveratrol therapy for 6 weeks. ResVida^®^ is the oral preparation used in the study, which contains pure trans-resveratrol with a purity of over 99% and is manufactured by DSM nutritional products. Healthy African American women will receive a daily dosage of 150 mg in the form of one capsule. The study involves four visits, including an initial visit and three additional visits at two-week intervals. The study coordinator will provide each participant with a two-week supply of a RSV and a medication calendar during visits 2–4. The blood samples will be collected by a trained phlebotomist during each visit and handed to the research laboratory to measure the IGF2 levels and other biomarkers. 

#### 3.4.4. Stilbenes and Prostate Cancer 

Paller et al. [150] conducted two studies to assess the effectiveness of low microgram doses of pulverized muscadine grape extract (MPX) capsules as a treatment option for patients with biochemically recurrent prostate cancer. The first study, which lasted for 28 days, evaluated the safety of both: a low dose (500 mg) and a high dose (4000 mg) of MPX. The second study lasted for 12 months [151], and despite treatment with microgram doses, the prostate-specific antigen doubling time (PSADT), which indicates disease progression, remained unchanged, indicating that the doses used were below the therapeutic threshold. Van Die et al. [152] also tested a blend containing a higher dose of resveratrol (30 mg) in a similar group of patients and reported an insignificant increase in PSADT. However, it should be noted that low doses were used in the studies, which may explain the lack of effect, and that much higher doses may be necessary for therapeutic efficacy. Additionally, the impact of the other constituents in the formulation on the pharmacokinetic profile of resveratrol is currently unknown. Further studies that use pure resveratrol at higher doses in a similar group of patients would be beneficial. 

#### 3.4.5. Stilbenes and Multiple Myeloma 

Popat et al. in 2009 conducted a clinical trial on the safety and activity of SRT501 (resveratrol) alone or in the combination with bortezomib in patients with multiple myeloma (MM). Unfortunately, the study has been terminated due to the extensive adverse effects (AEs) experienced by all of the 24 participants, including nausea (79%), diarrhea (71%), vomiting (54%), fatigue (46%), and anemia (38%). Adverse effects above or equal to grade 3 were reported by over half of the participants. Out of 24 patients treated with SRT501, 15 were withdrawn from the treatment. Eleven patients discontinued before the first response assessment and were not evaluable. The median time to progression was 2.8 months, and the overall survival was not reached. During the study, two deaths were reported—one possibly treatment-related and other one due to progressive disease. None of the patients treated with resveratrol monotherapy achieved a minor, partial, or complete response [153].

### 3.5. Carotenoids 

Carotenoids are a diverse class of colorful red, orange, and yellow terpenoid pigments found in fruit, vegetables, eggs, meats, milk, and some fish and crustacean seafoods [154]. Lycopene is present in brilliantly colored fruits and vegetables, such as ripe tomatoes, watermelon, beets, and red grapefruits [155]. β-Carotene is found in deep red and yellow vegetables and fruits and is responsible for the orange color of carrots. Green vegetables contain a high amounts of both the hydrocarbon carotenes and xanthophylls; capsanthine is responsible for the brilliant red pigment of peppers, and the pink/red coloration of crustaceans is due to the astaxanthin (ASTA).

**Figure 9:**
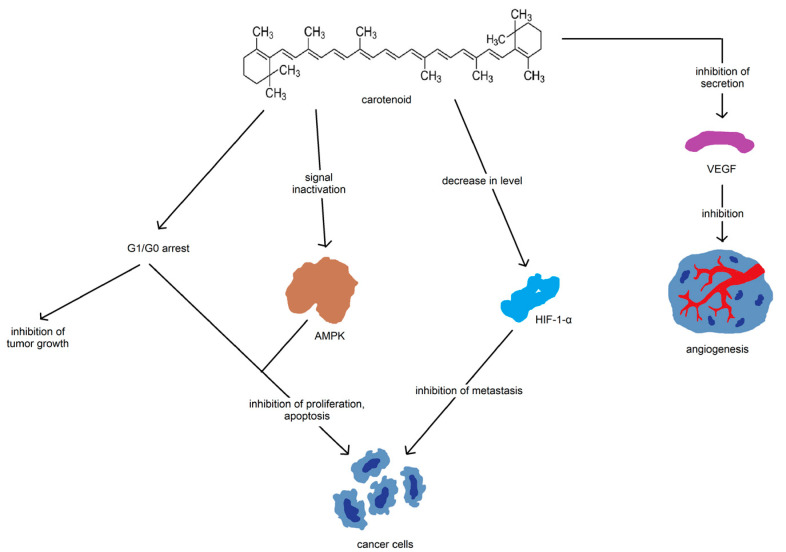
Anticancer mechanism of action of carotenoids.

Aside from their aesthetic properties, carotenoids are also potent antioxidants and are important for the production of nutrients, such as a vitamin A [156]. The major mechanisms through which carotenoids have been implicated in the cancer are related to the pathways involving cell growth and death [157]. Other pathways that are commonly modified by carotenoid consumption are related to their function as an antioxidant (Figure 9) [157]. 

There are more than 600 carotenoids with natural structural variants [158,159]. The main carotenoids include lycopene, β-carotene, ASTA, lutein, and zeaxanthin [160]. Carotenoids are synthesized by the linkage of two C20 geranylgeranyldiphosphate molecules. All carotenoids contain a polyisoprenoid structure, a longconjugated chain of double bonds, and near bilateral symmetry around the central double bond [161]. Carotenoids can be divided into provitamin A (e.g., β-carotene, α-carotene, γ-carotene, and β-cryptoxanthin) and non-provitamin A compounds [162]. 

On the other hand, carotenoids can be classified based on their functional groups as follows: (a) the xanthophylls (e.g., lutein, zeaxanthin) containing oxygen as a functional group, and (b) the carotenes (e.g., α-carotene, β-carotene and lycopene) containing only a parent hydrocarbon chain without any functional group [161]. 

A typical human diet contains around 40 carotenoids, and about 20 carotenoids are found in human blood and tissues, including β-carotene, α-carotene, lycopene, lutein, and cryptoxanthin [163]. Certain carotenoids can contribute to the body’s vitamin A requirements, support vision and epithelial cell regeneration, and control gene expression through the metabolite retinoic acid [164].

In a recent study [149], it was discovered that a carotenoid extract from *Dunaliella salina* containing lutein, zeaxanthin, α-carotene, and β-carotene inhibited the production of pro-inflammatory cytokines and enzymes, such as NO and COX-2, in LPS-activated cells. The extract exhibited anti-inflammatory properties through the inhibition of NF-κB activation and JNK phosphorylation [165]. Saffron extract, which is abundant in carotenoids, was found to decrease the viability of liver cancer cells in a dose- and time-dependent manner while also reducing inflammation, oxidative stress, and cell proliferation. It induced apoptosis and down-regulated inflammatory markers, such as COX-2, iNOS, and NF-κB-p65, in vivo [166]. 

Despite extended evidence on beta-carotene effectiveness on the molecular level, Hennekens et al.’s study on supplementation with 50 mg of β-carotene on alternate days in 11,036 male physicians found a lack of evidence on its effect on the incidence of malignant neoplasms and cardiovascular disease in comparison to that in 11,035 men in a control group throughout the 12-year study time [167]. Similar results were obtained in similar studies, including Lee et al.’s study on 19,939 women receiving 50 mg of beta-carotene on alternate days and 19,937 women in a control group during 2.1 years of treatment plus another 2.0 years of follow-up [168] and Connet and Prentice’s studies investigating carotenoid serum concentration levels [169,170]. 

#### 3.5.1. Carotenoids and Prostate Cancer 

The most examined carotenoids in terms of their importance in protection from prostate carcinogenesis and its progression are lycopene, β-carotene, and α-carotene [171]. Results in the most updated meta-analyses show that higher lycopene consumption and circulating blood concentrations are associated with a decreased risk of prostate cancer. In a meta-analysis of 42 studies that involved almost 700,000 participants, it was found that the risk of prostate cancer decreased by 1% for every additional 2 mg of lycopene consumed in the diet and by 4% for every additional 10 μg dL^−1^ of circulating lycopene [172]. Another meta-analysis from 2015 [173] showed that higher intake of α-carotene was associated with a 13% lower relative risk of prostate cancer, and each additional 0.2 mg of α-carotene consumed reduced the prostate cancer risk by 2%. However, there was no association found between the β-carotene and prostate cancer risk. Chemoprevention in prostate cancer has also been evaluated in the clinical trial by the National Taiwan University Hospital examining the effects of multi-carotenoid (MCS) supplementation, although no results has been posted and the study was last updated in October, 2015. The study was set to evaluate the effectiveness of the administration of 30, 15, or 0 mg of MSC orally daily in a group of 300 high-risk patients. Outcome measures include a comparison of the cumulative prostate cancer incidence among the groups and the change from baseline in the serum carotenoid levels. Previous Phase II and III clinical trials by the investigators reported no MCS-related serious adverse events (SAEs), although there is no information available on the AEs in this study [174].

#### 3.5.2. Carotenoids and Breast Cancer 

Only β-carotene, blood α-carotene, and blood lutein showed an association with a decrease in breast cancer risk. In a dose-response analysis, dietary β-carotene was associated with a 5% decrease in breast cancer for each additional 5 mg consumed, while blood α-carotene and β-carotene had a significant linear dose-response—breast cancer risk decreased by 26% for each additional 50 μg of β-carotene dL^−1^ or 18% for each additional 10 μg α-carotene dL^−1^. Additionally, breast cancer risk decreased by 32% for each additional 25 μg lutein dL^−1^ [175]. The Cancer Prevention Study II (CPS-II) Nutrition Cohort evaluated associations between plasma carotenoids and invasive breast cancer in post-menopausal women [176]—only plasma α-carotene was associated with invasive breast cancer, especially for estrogen receptor-positive breast cancer. The pre-diagnostic consumption of β-carotene was associated with a 30% decrease in all-cause mortality for the highest quantile and a 7% decrease in risk for 1.2 g per day for all-cause fatalities. No associations between lycopene, α-carotene, β-cryptoxanthin, or lutein consumption and all-cause mortality or breast-cancer specific survival have been found [177]. Rock et al.’s analysis of 3043 Women’s Healthy Eating and Living Study participants diagnosed with early-stage breast cancer revealed a greater likelihood of breast cancer-free survival regardless of the study group assignment correlation with higher biological exposure to carotenoids in the 6-year assessment time [178]. Kabat et al. established in his study on 5450 women that the risk of invasive breast cancer is inversely associated with alpha-carotene serum concentrations and positively associated with a lycopene [179]. 

#### 3.5.3. Carotenoids and Lung Cancer 

Patterns of smoking tend to be closely associated with less healthful diets, including lower consumption of fruits and vegetables [180,181]. A meta-analysis of 18 prospective studies found that higher blood concentrations of lycopene, α-carotene, and β-carotene were associated with a significant decrease in lung cancer risk [182]. Similarly, a study of 35 studies found that high consumption of lycopene, α-carotene, β-cryptoxanthin, and lutein/zeaxanthin were all associated with a significant decrease in lung cancer risk [183]. Notably, these associations were only observed in the current smokers. These associations were lost in individuals that were former smokers or individuals that never smoked [183]. However, high doses of β-carotene supplementation have been found to increase the lung cancer risk in current smokers and individuals with asbestos exposure [184,185]. These studies used much higher doses of β-carotene compared to consumption in a normal diet, which may have produced the pro-oxidant effects in the highly oxidative lung environment caused by the cigarette smoke. In the Beta-Carotene and Retinol Efficacy Trial (CARET) [184,186], a combination of 30 mg beta-carotene and 25,000 IU retinyl palmitate (vitamin A) taken daily was tested against a placebo in 18,314 men and women at a high risk of developing lung cancer. However, the intervention was stopped 21 months early due to the evidence of no benefit and possible harm. The active intervention group had 28% more lung cancers and 17% more deaths compared to the placebo group. The participants who received the combination of the beta-carotene and vitamin A did not experience any benefits and instead had a higher incidence and mortality rates from lung cancer. These results align with those of the Alpha-Tocopherol Beta-Carotene Cancer Prevention Study conducted with 29,133 male smokers in Finland, which also found no benefit from beta-carotene [187].

#### 3.5.4. Carotenoids and Gastrointestinal Cancers 

The consumption of the β-carotene was linked to a lowered chance of esophageal adenocarcinoma [188]. A meta-analysis on pancreatic cancer conducted before 2014 synthesized the available data, indicating that of the 18 studies examined on the correlation between dietary carotenoids and pancreatic cancer, lycopene, β-carotene, and β-cryptoxanthin exhibited a significant association with a decreased risk of pancreatic cancer, as reported in a reference [189]. In a 2013 study, comparing the highest and lowest quartiles of lycopene exposure, both dietary and blood lycopene were not found to be associated with gastric cancer, but an interesting finding was that increased tomato consumption was linked to a reduced risk of gastric cancer [190].

#### 3.5.5. Carotenoids and Colorectal Cancer 

A study was conducted starting in July 2010, which recruited 538 individuals with colorectal cancer and 564 controls who were matched for age (in 5-year intervals) and sex [191]. The study measured the levels of certain nutrients (α-carotene, β-carotene, β-cryptoxanthin, lycopene, and lutein/zeaxanthin) in the participants’ serum using HPLC. After adjusting for the various factors that could affect the results, the study found that there was an inverse relationship between the serum levels of α-carotene, β-cryptoxanthin, and lycopene and the risk of colorectal cancer. The adjusted odds ratios of the highest quartile of serum levels compared to the lowest quartile were 0.49 for α-carotene, 0.44 for β-cryptoxanthin, and 0.36 for lycopene. However, there was no significant statistical association between the serum levels of β-carotene and lutein/zeaxanthin and colorectal cancer risk. These findings suggest that lower levels of the α-carotene, β-cryptoxanthin, and lycopene in the serum are associated with a higher incidence of colorectal cancer in the Chinese population residing in Guangdong. Between July 2010 and October 2013, a study was conducted involving 845 individuals with colorectal cancer and 845 controls who were matched for the age and sex. Participants completed the in-person interviews, and their dietary intake was estimated using a validated food frequency questionnaire. The study found a strong inverse association between the intake of β-cryptoxanthin and the risk of colorectal cancer. Participants in the highest quartile of intake had a 77% reduced risk compared to those in the lowest quartile. Similarly, inverse associations were found for α-carotene, β-carotene, and lycopene. However, there was no statistically significant association between lutein/zeaxanthin intake and colorectal cancer risk. These findings were consistent across cancer sites, sources of controls, and smoking statuses. The study found that both males and females had inverse associations between the dietary intake of α-carotene, β-cryptoxanthin, and lycopene and colorectal cancer risk, while inverse associations between β-carotene intake and colorectal cancer risk were only observed in males. 

#### 3.5.6. Carotenoids and Head and Neck Cancer 

Meyer and Bairati conducted extensive research on the influence of nutritional compounds on head and neck cancer patients. Over the course of the clinical trial, investigators evaluated the relationship between α-tocopherol and β-carotene administration and the occurrence of secondary primary cancers, adverse effects of radiotherapy, and mortality. Supplementation consisted of a daily dose of vitamin E (1 capsule of 400 IU DL-α-tocopherol) and β-carotene (1 capsule of 30 mg) or placebos during the radiation therapy and for 3 years after the radiation therapy ended [192,193,194,195]. Among a clinical group of 273 patients with stage I or II squamous cell carcinoma of the head and neck treated with radiotherapy, a statistically significant increased all-cause mortality rate, with HR = 1.38 (95% CI = 1.03–1.85), was reported in comparison to that in the placebo group. The median duration of the radiation therapy was 43 days, and the median duration of the supplementation was 3.1 years. Overall survival was consistently lower among the participants randomized to the supplement arm (*p* = 0.033) [195]. During the supplementation period, the rate of a second primary cancers was statistically significantly higher among patients in the supplement arm (60 per 1000 person-years) than among patients in the placebo arm (25 per 1000 person-years), which became inverse after supplement discontinuation with 39 per 1000 person-years in the previous supplement users and 69 per 1000 person-years in the former placebo users [193]. A higher dietary intake of beta carotene was associated with approximately 40% lower frequencies of severe acute adverse effects of radiation therapy to the specific sites and overall. A similar pattern of inverse relationships was observed between the plasma beta carotene and severe acute adverse effects of radiation therapy [192,194]. 

Furthermore, lycopene, α-carotene, and β-cryptoxanthin were also found to be associated with a significant reduction in the incidence of oral and pharyngeal cancer [196]. 

#### 3.5.7. Carotenoids and Skin Cancer 

Lutein, by lowering the very-low-density lipoprotein (VLDL) and the intermediate density lipoprotein (IDL) levels, has been associated with a lower incidence of skin cancer [197]. In several long-term, large-cohort studies on the effect of the beta-carotene supplementation on the chemoprevention of skin cancer (basal cell carcinoma or squamous cell carcinoma), no significant evidence has been found [198,199,200]. 

In 1999, Green et al.’s study of 621 residents of Nambour, a township in southeast Queensland, analyzed skin cancer chemoprevention with daily sunscreen application and beta-carotene supplementation. An evaluation of the effect of beta-carotene supplementation was based on skin cancer occurring on any part of the body. After 4.5 years of follow-up, there was no difference in the incidence of basal-cell carcinoma in the beta-carotene and placebo groups. The incidence of SCC was slightly but not significantly higher in the beta-carotene group than in the placebo group [201]. 

A study by Heinen et al. was a further follow-up from 1996 until the end of 2004 of randomly selected Nambour Skin Cancer Study participants. The 8-year prospective study was evaluating all occurrences of BCC and SCC among the 1027 participants according to their dietary beta-carotene intake measured via a self-administered, semi-quantitative food frequency questionnaire consisting of 129 food or food group items. The BCC tumor risk was found to be double in persons with intake levels in the second tertile for β-carotene (multivariable adjusted RR = 2.2, 95% CI: 1.2–4.1). In the 191 participants with a history of BCC, the cancer risk was found to be equally as high (multivariable adjusted second tertile estimates for β-carotene: RR = 1.9, 95% CI: 0.97–3.6, P for trend = 0.75, and vitamin E: RR = 2.4, 95% CI: 1.3–4.7, P for trend = 0.20) [197]. 

In the clinical trial ‘Correlation Between Skin Carotenoid Levels and Previous History of Skin Cancer’, Alexandra Kimball from Massachusetts General Hospital investigated the skin beta-carotenoid levels in relation to the skin cancer status in groups with a history of BCC or SCC and a control group without a skin cancer history. No difference in the mean carotenoid levels between subjects in the investigation groups and control subjects was found [202]. 

#### 3.5.8. Carotenoids and Gynecological Cancers 

Similarly, dietary lycopene was not associated with ovarian cancer [203], but a previous meta-analysis from 2001 showed that higher consumption of β-carotene was associated with a 16% reduction in the ovarian cancer risk [189].

Most studies that have investigated the relationship between cervical cancer risk and dietary habits, focusing on vitamin A or plasma/serum retinol concentrations, have not found any significant association [204,205,206,207,208,209]. However, in the studies where carotenoids, carotenoid-rich foods, or plasma/serum concentrations of these compounds have been examined, carotenoids have been linked to cervical cancer risk and progression [204,205,206,207,208,209,210]. Low intake or serum levels of carotenoids appear to increase the risk of the cervical carcinoma. Moreover, lower blood levels are associated with an increased risk of persistent cervical HPV infection [211]. Studies that have examined individual carotenoids, such as beta-carotene, lycopene, alpha-carotene, and beta-cryptoxanthin, have suggested that they may provide a protective effect [212,213]. The topical application of all-trans-retinoic acid, a metabolite of vitamin A and provitamin A carotenoids, has been shown to enhance the regression of cervical intraepithelial neoplasia (CIN) II in clinical trials [214]. Several chemoprevention trials utilizing beta-carotene supplements to promote the regression of cervical dysplasia have been conducted [204,214], but some have had negative results [215,216]. A randomized clinical study in the Netherlands [215], where almost 280 women with CIN were dosed with 10 mg b-carotene or placebo daily for 3 months, takes note of no effects on regression observed. Romney et al. [217] also reported no effects of supplementation in a double-blind trial involving 30 mg b-carotene or placebo administered daily to 98 patients with CIN who were followed for 9 months. However, a nonrandomized phase II study of women with CIN I and CIN II [218] showed an improvement in 70% of cases after 6 months of daily supplementation with 30 mg beta-carotene, and these findings are being studied further in a randomized phase III trial [219]. Three randomized controlled trials in Australia [220] were also investigating the effects of beta-carotene supplementation in women with lower grade cervical dysplasia—two of them are examining the effect of 30 mg b-carotene plus 500 mg vitamin C and 15 mg b-carotene plus 300 mg vitamin C among women with CIN I and the other one involves the administration of 30 mg b-carotene/day specifically to assess HPV changes over a 2-year period.

#### 3.5.9. Carotenoids and Other Types of Cancers 

Each additional 1 mg of α-carotene intake corresponded to a 13% reduction in the risk of non-Hodgkin Lymphoma, according to the findings reported in a reference [221]. 

## 4. Discussion 

Phytochemicals consist of a wide arrange of the chemical compounds present in a variety of plants, including the flavonoids, phytosterols, phenolic acids, stilbenes, and carotenoids described in our work. Due to the nature of their origin, it is almost impossible to assess the role of the singular compounds in human studies as they are consumed mostly in a form of fruits and vegetables filled with an abundance of the different phytochemicals, of which it is almost impossible to discern their influence on the human body.

**Figure 10:**
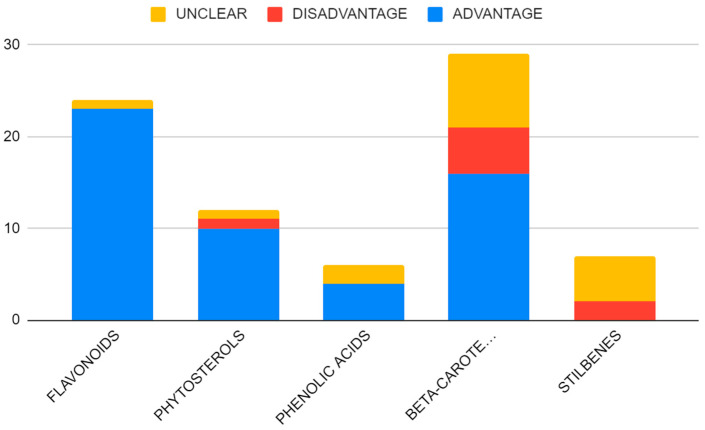
Advantage analysis of the study results based on the compound researched.

The majority of the epidemiological studies report a significant advantage of heightened phytochemical consumption and increased phytochemical serum levels, linking increased exposure of these compounds with a lower cancer risk across the majority of cancer types, which can be demonstrated by the flavonoid group. None of the studies on flavonoids were interventional, simultaneously achieving the greatest advantage among the depicted subgroups. Nonetheless, most supplementation studies were conducted to investigate beta-carotene potential—a group with the largest count of the results showing disadvantage. (Figure 10). Similarly, all studies including stillbenes were interventional and none of them showed any advantage (Figure 10). It is also possible for certain compounds to be more beneficial in their anticancer mode of action, although there is still a distinctive lack of research in this area to evaluate this statement. 

Such results strongly corelate with in vivo studies, where multiple plant-derived substances were found to have an anticancer and chemopreventive mode of action based on a variety of different mechanisms, including antioxidant, anti-inflammatory, induction of apoptosis, detoxification, cellular damage prevention, inhibition of cell proliferation, inhibition of cell cycle progression, inhibition of cell invasion, migration, and adhesion, and stimulation of the immune function [3,4,5,6,7,8,9,17,18,19,20,21,22,38,39,40,41,42,43,44,45,46,47,48,49,78,106,107,108,109,110,111,112,113,114,115,116,117,118,119,120,124,134,135,136,137,138,139,140,141,142,157]. Unfortunately, these effects could not be replicated in most available clinical trials, many of which were withdrawn early due to a lack of evidence and/or risk of harm (Table 3).

Several completed studies reported an increased cancer risk and heightened mortality in a clinical arm receiving phytochemical supplementation (Table 4). Moreover, no universal correlation among the dose intake, effectiveness, and adverse effect severity can be established as the results between individual studies vary greatly. Considering the small therapeutic effect, as in the vast majority of cases the noted advantage was not statistically significant, high concern can be implemented on the safety of future human phytochemical studies due to the high-risk adverse effect profile and small clinical advantage reported in available studies. This concern also concerns a possible need for the reformulation of the phytochemical compounds in human studies.

Most of the concentrations used in in vitro studies are ineffective in clinical application due to the low bioavailability of the phytochemicals [119,126,130]. It is, therefore, possible, that a clinically active dose may lead to overexposure of the compound in certain tissues and cause severe adverse effects, reported in particular clinical trials, including multiple myeloma and CARET studies [153,184,185,186]. 

**Figure 11:**
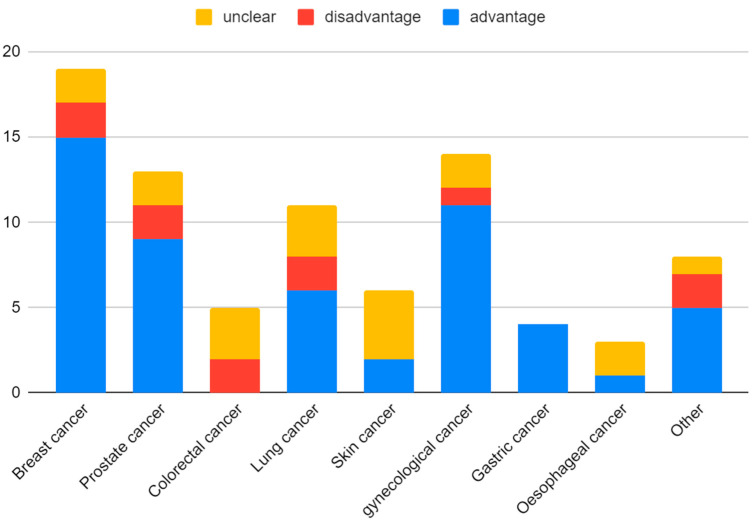
Advantage analysis of the study results based on the cancer subtype researched. Note: Other cancers included: head and neck cancers (2), pancreatic cancer (3), urinary bladder cancer (1), multiple myeloma (1), and neuroendocrine cancer (1).

Although the estimated share of the advantageous to the disadvantageous results seems to be similar among the various cancer subtypes, it is possible, however, to pinpoint the group of neoplasms that are extremely unfavorable in phytochemical supplementation. (Figure 11) Multiple myeloma, non-melanoma skin cancer, and lung cancer belong to the cancer subgroups with the most concerning results, such as a heightened risk of the neoplasm or severe, possibly fatal adverse effects [153,184,185,186,197,198,199,200,201,202]. Most positive results from the phytochemicals were depicted in breast and gynecological tumors, what may suggest an important role of the phytochemicals in women’s hormone imbalances.

It is worth noting that the best results of chemoprevention were described in the cohorts with a phytochemical-rich diet, consisting mostly of a plant-based food. Therefore, there is a possibility that not a singular phytochemical is responsible for the anticancer mode of action, but rather the cluster of various substances available organically in plants. Diet-based phytochemical intake allows for a balance of the vast variety of compounds and to avoid overexposure to a singular substance. 

Several clinical trials involving phytochemicals are currently being conducted (Table 4). Additional trials, conducted with great care related to the safety profile, with a larger sample size and sufficient follow-up time, are needed to further elucidate the role and associations of phytochemicals in cancer patients. 

## 5. Conclusions 

The strong anticancer effect of phytochemicals is well-established and proven by multiple epidemiological studies in the several cancers, mostly based on a plant-rich diet. The great anticancer potential observed in the variety of in vitro studies cannot be, however, replicated in vivo in humans due to the toxicity and bioavailability limitations. The great concerns caused by the worrisome results, including an increased cancer risk in case cohorts, implies an urging need for further research. Although human studies and clinical trials are greatly needed, they must be conducted with great caution regarding the safety measures. 

## Figures and Tables

**Table 1:** Number of studies described in the compound-specific subsection.

Compound	Number of Studies
Flavonoids	24
Phytosterols	9
Phenolic acids	6
Beta-carotenoids	27
Stilbenes	7

**Table 2:** Case and control cohort sizes.

Substance	Case Cohort	Control Cohort
Flavonoids	556,799	245,864
Phytosterols	162,617	4378
Phenolic acids	11,327	222
Beta-carotenoids	3,778,821	27,735
Stilbenes	930	1440

**Table 3:** Clinical trials on the prementioned phytochemicals.

Trial Number	Years	Location	Condition (Only Significant for Article)	Number Recruited	Substance	Outcome Measure	Status
NCT00712647	05.1985–06.2005	United States	Lung Cancer	18,314	Beta Carotene, Retinol	Prevention Of Lung Cancer	Completed
NCT00003094	04.2004–05.2014	United States	Cervical Cancer	0	Preventative Dietary Intervention	Carotenoid Level; Progression Of Cervical Dysplasia	Withdrawn *
NCT00098969	12.2004–12.2012	United States, United Kingdom	Unspecified Adult Solid Tumor, Protocol Specific	40	Resveratrol	Concentration Of Resveratrol and its Metabolites in the Plasma, Urine, and Feces; Determination of the Drug Safety in Participants	Completed
NCT00169845	09.2005–03.2018	Canada	Head and Neck Neoplasms	540	Alpha-Tocopherol; Beta-Carotene	Prevention of Second Primary Cancers	Completed
NCT00256334	06.2005–04.2009	United States	Colon Cancer	11	Resveratrol	Expression of a Panel of Wnt Target Genes	Completed
NCT00342992	06.2006–09.2020	Finland	Cancer	29,133	Alpha-Tocopherol; Beta-Carotene	Annual Linkage with Finnish Cancer Registry	Completed
NCT00578396	12.2007–01.2021	United States	Colon Cancer	0	Grapes	Localization of β-Catenin and Wnt Target Gene Expression in Intestinal Mucosa	Withdrawn **
NCT00433576	02.2007–09.2014	United States	Adenocarcinoma of the Colon Adenocarcinoma of the Rectum Colon Cancer (Stafe I–III) Rectal Cancer (Stage I–III)	20	Resveratrol	Pharmacodynamics of Resveratrol; Concentrations of Biomarkers	Completed
NCT00712647	07.2008–09.2012	United States	Lung Cancer	18,314	Beta Carotene; Retinol	Lung Cancer Incidence [bi-annual ]	Completed
NCT00836342	02.2009–06.2012	United States	Skin Cancer Basal Cell Carcinoma Squamous Cell Carcinoma	81	Skin Carotenoid	Skin Carotenoid Levels	Completed
NCT00920556	03.2009–11.2010	Denmark, United Kingdom	Multiple Myeloma	24	SRT501, Bortezomib	Safety Assessments	Terminated
NCT01476592	11.2011–10.2013	United States	Neuroendocrine Tumor	7	Resveratrol	Notch1 Activation in Tumor Biopsy Specimens	Completed
NCT01692340	09.2012–03.2019	United States	Healthy	12	Lycopene, Phytoene, Phytofluene	Plasma Half-Life of Labeled Carotenoid; Maximal Plasma Carotenoid Concentration; Time of Maximal Carotenoid Concentration	Completed
NCT01538316	02.2012–05.2012	Germany	Primary Prevention of Prostate Cancer	60	Quercetin; Genistein	Log2-Transformed PSA Measurements	Unknown
NCT02261844	10.2014–03.2017	United States	Liver Cancer	0	Resveratrol	Improvement of the Metabolic Profile of Liver Cells	Withdrawn ***
NCT02426216	04.2015–10.2015	Taiwan	Prostate Cancer	300	Multi-carotenoids	Cumulative Histologically Proven Prostate Cancer Incidence at 2 years	Unknown
NCT03433092	11. 2017–12.2017	China	Gastrointestinal Tract Cancer	40,641	Serum Carotenoids	Serum Carotenoid Levels; Relative Risk (RR) or OR and Corresponding 95% CI	completed
NCT03070262	01.2017–12.2021	China	Esophagus Cancer	300	Caffeic Acid	1-Year Overall Survival (OS)	Unknown
NCT04648917	05. 2019–05.2022	China	Esophagus Cancer, Stage III	80	Caffeic Acid	1-Year Overall Survival (OS)	Unknown
NCT04266353	02.2020–05.2022	United States	Chemoprevention	50	Resveratrol	Serum/plasma IGF-II/IGFBP-3 Concentrations	Suspended
NCT04147767	02.2020–12.2021	United Kingdom	Breast Cancer	50	Dietary Supplement: Cholesterol Reducing Yogurt Drink	Serum/Plasma Oxysterol Concentrations	Recruiting

* (Study was halted prematurely, prior to enrollment of first participant), ** (Non applicable clinical trial), *** (No funding).

**Table 4:** Results of the intervention studies involving compound supplementation.

Substance	Additional Substances	Time Interval	Dosage	Outcome
**Phytosterols**				
Phytosterols		20 weeks	2 g/d free plant sterols	Results not available [97]
		6 years	1 L/week of extra virgin olive oil	62% relatively lower risk of malignant breast cancer [95]
		6 years	30 g/d of mixed nuts	Nonsignificant breast cancer risk reduction [95]
**Phenolic acid**				
Caffeic acid		1 year	100–200 mg/d, depending on body mass	Results not available [118]
		1 year	300 mg/d	Results not available [119]
** Beta-carotene**				
Beta-carotene		8 years	20 mg/d	Inverse association between dietary intake of alpha-tocopherol and beta carotene and the risk of lung cancer; higher mortality among recipients of beta carotene [187]
		8 years	20 mg + 50 mg/d of alpha-tocopherol	
	beta-cryptoxanthin, lutein, lycopene	5 years	daily intake of 5 vegetable servings, 16 oz of vegetable juice or vegetable equivalents, 3 fruit servings, 30 g fiber, and 15–20% energy intake from fat	21% relatively lower risk of malignant breast cancer [178]
		9 months	30 mg/day	Lack of beneficial effect on; CIN (cervical intraepithelial neoplasia) [217]
		12 months	30 mg/day	70% response rate of CIN at 6 months and 43% at 12 months [218]
		5 years	30 mg/day + and a sun protection factor 15+ sunscreen	Slightly but not significantly lower incidence for basal-cell carcinoma (BCC); slightly but not significantly higher incidence of squamous-cell carcinoma (SCC) [201]
			15 mg + 300 mg vitamin C	
			30 mg/day	
	vitamin C	6 months	15 mg + 500 mg vitamin C	Results not available [220]
		12 months	30 mg	
	alpha tocopherol	3 years	30 mg + 400 IU of alpha-tocopherol	Fewer severe acute adverse effects [139]
		2 years	30 mg + 25,000 IU retinil palmitate	Lack of chemopreventive benefit and excess lung cancer incidence and mortality [184,185,186]
		5 years	50 mg/day	Lack of beneficial effect on the rate of occurrence of first new non-melanoma (NMSC) skin cancer [198]
		12 years	50 mg on alternate days	Lack of beneficial effect on first NMSC, including BCC and SCC, prevention [200]
** Stilbenes**				
Resveratrol SRT501		3 months	5 mg/day in 2 divided doses of 2.5 mg	Results not available [148]
	bortezomib	3 years	5 g/day	Unacceptable safety profile and minimal efficacy in patients with relapsed/refractory MM [153]
		2 weeks	20, 80, 160 mg/day + grape extract 125 mg/day + 8 oz glass of water	Inhibitory effect on Wnt signal throughput in colonic mucosa-derived cell line [146]
	Curcumin, catechins, fresh borccoli sprouts	12 weeks	curcumin 100 mg/d; resveratrol 30 mg/d; catechins 100 mg/d; fresh broccoli sprouts equivalent to 2000 mg/d	Non-significant increase in the log-slope of PSA in the active treatment group [151]
		6 weeks	150 mg/day	Results not available [149]
MPX (Muscadine Grape Skin Extract)		1 year	500 mg/day to 4000 mg/day	No severe related adverse events were reported [149]
		1 year	4000 mg/d of MPX, 500 mg/d of MPX (1.2 mg of ellagic acid, 9.2 μg of quercetin, and 4.4 μg of trans-resveratrol)	No significant difference in PSADT change [150]
		1 year	1.2 mg of ellagic acid, 9.2 μg of quercetin, and 4.4 μg of trans-resveratrol	

## Data Availability

Not applicable.
